# Impact of COVID-19 restrictive measures on income and health service utilization of tuberculosis patients in India

**DOI:** 10.1186/s12879-022-07681-z

**Published:** 2022-08-29

**Authors:** Susmita Chatterjee, Palash Das, Anna Vassall

**Affiliations:** 1 George Institute for Global Health, Research Department, 308, Elegance Tower, Plot No 8, Jasola District Centre, 110025 New Delhi, India; 2 Department of Medicine, University of New South Wales, 18 High Street, 2052 Kensington, NSW Australia; 3 Prasanna School of Public Health, Manipal Academy of Higher Education, Madhav Nagar, 576104 Manipal, Karnataka India; 4 London School of Hygiene and Tropical Medicine, Department of Global Health and Development, 15-17 Tavistock Place, WC1H 9SH London, UK

**Keywords:** COVID-19, Economic impact, Employment, Financial Impact, Income loss, Informal sector, Tuberculosis, India

## Abstract

**Background:**

The nationwide lockdown (March 25 to June 8, 2020) to curb the spread of coronavirus infection had significant health and economic impacts on the Indian economy. There is limited empirical evidence on how COVID-19 restrictive measures may impact the economic welfare of specific groups of patients, e.g., tuberculosis patients. We provide the first such evidence for India.

**Methods:**

A total of 291 tuberculosis patients from the general population and from a high-risk group, patients from tea garden areas, were interviewed at different time points to understand household income loss during the complete lockdown, three and eight months after the complete lockdown was lifted. Income loss was estimated by comparing net monthly household income during and after lockdown with prelockdown income. Tuberculosis service utilization patterns before and during the lockdown period also were examined. Household income loss, travel and other expenses related to tuberculosis drug pickup were presented in 2020 US dollars (1 US$ = INR 74.132).

**Results:**

26% of households with tuberculosis patients in tea garden areas and 51% of households in the general population had zero monthly income during the complete lockdown months (April–May 2020). Overall income loss slowly recovered during July–August compared to April–May 2020. Approximately 7% of patients in the general population and 4% in tea garden areas discontinued their tuberculosis medicines because of the complete lockdown.

**Conclusion:**

Discontinuation of medicine will have an additional burden on the tuberculosis elimination program in terms of additional cases, including multidrug resistant tuberculosis cases. Income loss for households and poor restoration of income after the lockdown will likely have an impact on the nutrition of tuberculosis patients and families. Tuberculosis patients working in the informal sector were the worst affected group during the nationwide lockdown. This emphasizes that a policy priority must continue to protect those working in informal sectors from the economic consequences of such restrictive measures, including paid sick leave, additional food support, and direct benefit transfers. Alongside ensuring widespread access to COVID-19 vaccines, these policy actions remain pivotal in ensuring the well-being of those who are unfortunate enough to be living with tuberculosis.

**Supplementary Information:**

The online version contains supplementary material available at 10.1186/s12879-022-07681-z.

## Background

COVID-19 has severely impacted tuberculosis (TB) diagnosis and treatment in India. There was an approximately 80% decline in daily TB notifications during the nationwide lockdown period (from end March 2020 to June 2020) compared to average daily notifications [1]. Furthermore, in June 2020, the number of inpatient TB cases was one-third of the number in June 2019. In a civil society-led survey conducted to understand the impact of COVID-19 on TB, 85% of civil society and advocates interviewed in the survey agreed that there was a significant decrease in TB testing during the lockdown period in India [2]. Explanations of this decline included the following: GeneXpert machines and other laboratory facilities/space were switched for use for COVID-19 response, reduced availability of health care staff generally or because they were too busy with COVID-19 testing, lack of access to health facilities due to no access to public transport, and fear of getting infected with COVID-19 [1]. A recent modeling study estimated that there will be an increase of 182,000 TB cases and 83,600 deaths between 2020 and 2025 because of COVID-19 in India [3].

COVID-19 not only impacts the health sector response to TB and other diseases but also has a widespread economic impact. A large telephone survey conducted among 47,000 households across 15 states in India estimated that 55 million workers above the poverty line had lost their jobs temporarily or permanently during this crisis [4]. Another survey conducted among 5,000 households during and after the lockdown across six Indian states found that 30% of the sampled households had at least one sign of food insecurity, which included reduced meal size, ran out of food, hungry but did not eat or went without eating for the whole day [5].

Previous studies have modeled the potential impact of COVID-19 on TB and other services, such as childhood immunization [6]. However, there is limited empirical evidence on how COVID-19 restrictive measures may impact the economic welfare of a specific group of patients. We provide the first evidence of impact of COVID-19 nationwide lockdown on income and health service utilization of the TB patients in India, the disease that contributes the highest burden in the world, with an estimated incidence of 2.64 million in 2019 and approximately 450,000 deaths [7].

## Methods

### Study participants

This analysis is part of an ongoing cohort study that originally aimed to report the economic burden of TB in India using a representative sample of 1536 drug-susceptible TB patients from four states covering patients from the general population and from high-risk groups (socially vulnerable and clinically high-risk as identified in the national strategic plan for TB elimination 2017–2025, [8]). The details of the main study population, sample size calculation, patient recruitment and interview process are presented in Additional file 1: Annexure. This study presents the impact of COVID-19 restrictive measures on income for the families of TB patients and TB service utilization patterns during the nationwide lockdown period, which occurred in 291 study participants (89 from the general population and 202 from tea garden areas) during the continuation phase of their treatment^1^ in one state.

In the main study, all recruited TB patients were interviewed three times: at the intensive phase of treatment (0–2 months), at the continuation phase (5–6 months) and approximately one year posttreatment. In one of the study states, intensive phase interviews were conducted from January–February 2020, continuation phase interviews were conducted from July–August 2020 and posttreatment follow-up interviews were conducted from January–February 2021. Hence, the participants from that state were impacted by the COVID-19 restrictive measures that started with a stringent nationwide lockdown from March 25, 2020, until June 8, 2020, followed by several restrictions for a few more months, such as nonavailability of public transport and nonavailability of health services other than COVID-19 in many government health facilities, which disrupted general health services, including TB services.

### Impact of COVID-19 restrictive measures on household income

We examined the trend in net monthly household income of the sampled TB patients at different time periods (before TB, during the intensive and continuation phases of TB treatment and during the nationwide lockdown) to understand the impact of TB disease as well as the nationwide lockdown on household income. During intensive phase interviews, patients were asked to retrospectively report monthly net household income before the patient started TB treatment and at the time of the intensive phase interview. Similarly, during the continuation phase interviews, they were asked to retrospectively report net household income just before the lockdown started (February 2020), during the complete lockdown period (April and May 2020) and at the time of the interview (July and August 2020).

Monthly household income during the lockdown (April and May 2020) and during the interviews (July and August 2020) were subtracted from prelockdown monthly household income (February 2020) to estimate any income loss of the household having TB patients during these two time points: the beginning of April until the end of May 2020 and the beginning of April until the date of the interview in July–August 2020.

The patients were followed up in January–February 2021, which allowed us to assess whether these households recovered, and they were asked to report their household income during the time of the interview. Household income reported during the interviews in January–February 2021 was compared with household income reported in February 2020 (before lockdown) to understand the proportion who managed to return to the pre-COVID situation.

### Impact of COVID-19 restrictive measures on TB service utilization

To understand TB service utilization during the COVID-19-related lockdown, we adapted our original TB interviews (which are broadly in line with the World Health Organization TB patient cost survey instrument used in many countries) [9]. We added questions about the frequency, travel time and travel expenses related to TB drug pickup in the prelockdown and during lockdown periods, the discontinuation of TB medication (if any) because of the complete lockdown, and the number of days of the discontinuation of TB medicine to the continuation phase interviews.

#### (A) changes in TB drug pickup pattern in pre- and during lockdown period

To understand any change in the pattern of TB drug pickup because of the complete nationwide lockdown, patients were asked about the frequency of visits to get drugs, time spent for travel and drug collection, and travel and other expenses related to drug collection for two time periods—before the lockdown and during the lockdown. The lockdown period was considered from March 25, 2020, until the end date of TB treatment for each patient. The number of days from March 25, 2020, until the end of TB treatment was calculated for each patient, and the same number of days was applied before the lockdown phase to understand any difference in the frequency of drug pickup between the two time periods. Any difference in frequency of drug pickup between the two time periods had impact on time cost as well as other expenses related to drug pickup such as travel expenses. The time cost for TB drug pickup for the patient, household member and accompanying person (if any) in the prelockdown and during lockdown period was calculated by multiplying the total hours spent for drug pickup with the hourly wage and was compared between the two time periods. Hourly wages were estimated using the minimum wage rate of the study state. Travel expenses for TB drug pickup from March 25, 2020, till the end of TB treatment were compared with the travel expenses incurred for the same number of days in the prelockdown period.

#### (B) discontinuation of TB medicine during lockdown

To examine any discontinuation of TB medicine during the lockdown, patients were asked whether they missed the medicine because of the lockdown and, if yes, for how many days they missed the TB medicine.

The number of patients interviewed at different time points and for measuring different impacts are presented in Table 1.

**Table 1:** Number of TB patients included in the study

	Continuation phase interviews (Timeline: July–August 2020)	
	General population	Tea garden families
Number of TB patients interviewed	89	202
*Financial impact*		
Household income loss during complete lockdown (April-May 2020) and from April 2020 till the date of interview in July-August 2020	89	202
*Service utilization*		
Drug pick-up pattern from March 25, 2020, till the end of TB treatment and same number of days before lockdown	72	146
Medicine discontinuation (if any) from March 25, 2020, till the end of TB treatment	88	202
*Post treatment interviews (Timeline: January – February 2021)*		
Number of TB patients interviewed	101	240
*Financial impact*		
Household income loss from April 2020 till the date of interview in January-February 2021	84	193

(1) Number of TB patients interviewed in post treatment period (101) is higher than patients interviewed in continuation phase (89) because of loss to follow up in continuation phase but followed up in post treatment period. As continuation phase interviews was over phone, there were loss to follow up because of non-availability through phone. (2) Difference in number of TB patients interviewed for household income loss from April 2020 till January - February 2021 (84) as compared to patients interviewed in post treatment follow-up (101) is because of availability of household income data for both continuation phase and post treatment interviews. (3) Number of patients covered under drug pick-up are less than the number covered in continuous phase interview as some were under daily directly observed treatment (DOT) and pickup was not applicable. (4) Sample size for medicine discontinuation of general population is less as one patient defaulted and stopped medicine before lockdown started

### Data collection

All rounds of interviews were conducted by four trained research assistants using structured pretested questionnaires. The intensive phase and the posttreatment follow-up interviews were face-to-face, while the continuation phase interviews were conducted by telephone, as interstate travel was restricted. All interviews, including phone interviews, were attended by two core team members to ensure quality data. Household income loss, travel and other expenses related to TB drug pickup were presented in 2020 US dollars (US$). An average exchange rate of 1 US$ = INR 74.132 was used throughout the paper.

## Results

### Characteristics of study participants

In one of the study states, intensive phase interviews were conducted from January–February 2020 and covered 121 patients from the general population and 279 from tea garden areas. The participants from this study state were impacted by the COVID-19 restrictive measures during their continuation phase of treatment. Continuation phase interviews (July–August 2020) covered a total of 291 TB patients, of whom 89 were from the general population and 202 were from tea garden families. The reasons for the loss to follow-up during the continuation phase interviews are presented in Table 2. The characteristics of the patients interviewed over the phone during the continuation phase of treatment (July–August 2020) are presented in Table 3. The majority of patients were male and of younger age groups. A total of 17–47% of patients never attended school, and most of those among the general population who were in the job market before TB were engaged in the informal sector, i.e., either as contractual workers or daily wage earners. For the patients in tea garden families, 36% were in formal employment (i.e., permanent tea garden workers), and 34% were in the informal sector-either as contractual tea garden workers or daily wage earners.

**Table 2:** Reasons of loss to follow up

	Intensive phase – N (January-February 2020)	Continuation phase – N (July-August 2020)	Reasons – N (%)
General population	121	89	Death – 5 (4.13%) Refusal – 6 (4.96%) No trace – 4 (3.30%) Could not be contacted over phone – 17 (14.05%) Total – 32 (26.45%)
Tea garden families	279	202	Death – 20 (7.17%) Shifted to MDR – 4 (1.43%) No trace – 4 (1.43%) Migrate out – 1 (0.36%) Cancelled because of misdiagnosis – 1 (0.36%) Could not be contacted over phone – 47 (16.85%) Total – 77 (27.60%)

*MDR* multidrug resistant TB

**Table 3:** Characteristics of study participants

	Continuation phase (July–August 2020)	
	General population (N = 89)	Tea garden families (N = 202)
*Age*		
18–24 years	21 (23.60)	53 (26.24)
25–34 years	23 (25.84)	54 (26.73)
35–44 years	22 (24.72)	52 (25.74)
45–54 years	14 (15.73)	23 (11.39)
55–64 years	6 (6.74)	17 (8.42)
65 years and above	3 (3.37)	3 (1.49)
*Gender*		
Male	64 (71.91)	114 (56.44)
Female	25 (28.09)	88 (43.56)
*Education*		
Not attended school	15 (16.85)	94 (46.53)
Primary	41 (46.07)	86 (42.57)
Secondary	12 (13.48)	13 (6.44)
Higher secondary	11 (12.36)	6 (2.97)
Graduate	10 (11.24)	3 (1.49)
Post-graduate and above	0 (0.00)	0 (0.00)
*Employment status before TB*		
Unemployed	7 (7.87)	15 (7.43)
Formal paid work	12 (13.48)	72 (35.64)
Informal paid work	33 (37.08)	69 (34.16)
Retired	2 (2.25)	3 (1.49)
Student	5 (5.62)	9 (4.46)
Homemaker	12 (13.48)	25 (12.38)
Self-occupied	18 (20.22)	9 (4.46)
*Type of TB*		
Pulmonary, bacteriologically confirmed	39 (43.82)	104 (51.49)
Pulmonary, bacteriologically unconfirmed	14 (15.73)	43 (21.29)
Extra pulmonary	36 (40.45)	55 (27.23)

Figures in parentheses indicate percentage

Posttreatment interviews conducted in January–February 2021 covered 101 participants from the general population and 240 from tea garden families with similar characteristics to those found among participants in the continuation phase, as this group comprised those interviewed during the continuation phase plus those who were missed during the continuation phase interviews.

### Changes in pattern of monthly household income over time

**Fig. 1:**
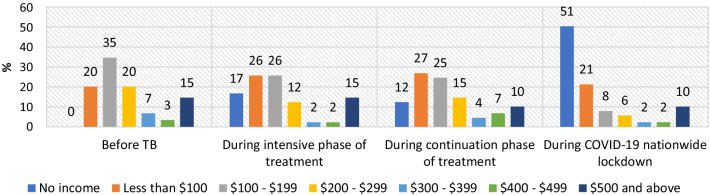
Percentage of households in different income groups over time: average monthly household income in 2020 US$ (patients in general population interviewed during July–August 2020, during continuation phase of treatment, N = 89)

Before a household member contracted TB disease, no household in the general population had zero household income (Fig. 1). Most of the households (35%) had monthly incomes in the range of US$100–US$199. However, the situation changed when a member of the household contracted TB. During the intensive phase of TB treatment of the household members, 17% of households had zero monthly income. The situation improved during the continuation phase of treatment, when 12% of households had zero income. The COVID-19 restrictive measures changed the situation drastically, and 51% of households had zero monthly income during the lockdown months (April–May 2020) (Fig. 1). Even if the effect of TB disease on household income is deducted (17% of households with zero income during the intensive phase and 12% during the continuation phase) from the proportion of households having zero income during the lockdown months (51%), a significant proportion of households were still impacted by the COVID-19 restrictive measures.

**Fig. 2:**
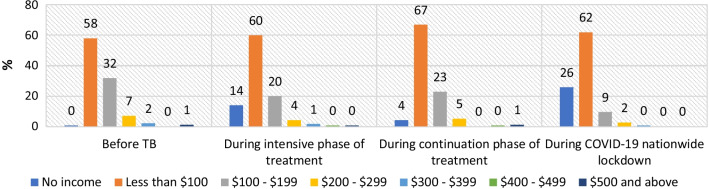
Percentage of households in different income groups over time: average monthly household income in 2020 US$ (patients in tea garden families interviewed during July–August 2020, during continuation phase of treatment, N = 202)

Similar trends were observed for the households in tea garden areas. 26% of the households of TB patients in tea garden areas had no income during the complete lockdown months, and there was a significant decline in the proportions of households in all other income ranges (Fig. 2).

### Household income loss over time

We report in Table 4 average household monthly income at different time points as well as income losses during two time periods: during the complete lockdown (April–May 2020) and from April 2020 until the date of the interview in July–August 2020. Overall income loss slowly recovered for the general population during July–August compared to April–May 2020; however, 8 months after the complete lockdown (January–February 2021), 44% of households in the general population and 37% of those from tea garden areas still suffered income losses when we compared their income during January–February 2021 with their prelockdown income (February 2020) (Not reported in table).

**Table 4:** Average monthly household income and income loss at different time points (2020 US$)

	Number	Average monthly income before lockdown (February 2020) - US$, 95% CI (LB, UB)	Average monthly income during lockdown (April-May 2020) - US$, 95% CI (LB, UB)	Average monthly income at the time of interview (July-August 2020) - US$, 95% CI (LB, UB)	Average per household income loss during April- May 2020 - US$, 95% CI (LB, UB)	Average per household income loss from April-August 2020 - US$, 95% CI (LB, UB)
General population	89	202.59 (159.73, 245.46)	114.44 (72.81, 156.07)	161.73 (118.83, 204.63)	(−) 220.39 (− 273.14, − 167.63)	(−) 153.96 (− 228.37, − 79.55)
Tea garden families	202	85.95 (75.97, 95.93)	47.21 (39.89, 54.53)	88.20 (78.99, 97.42)	(−) 96.85 (− 118.43, − 75.28)	11.70 (− 16.37, 39.77)

CI, confidence interval; LB, lower bound; UB, upper bound

### TB drug pickup before and during lockdown (continuation phase of treatment)

The TB drug pickup pattern for all patients was examined during the continuation phase of their treatment. A total of 72 patients from the general population and 146 from tea garden areas were covered for this analysis, as the rest covered during the continuation phase interviews were in daily directly observed treatment (DOT), and drug pickup was not applicable for them. Generally, during the lockdown period, the number of visits for drug pickup was lower for both groups, and consequently, total hours spent, and time cost were lower when compared with the prelockdown period. However, for the patients in tea garden areas, total expenses related to drug pickup (travel expenses and other expenses on food or payment related to drug pickup) were higher during the lockdown period, probably because of the remote locations of the tea gardens and the nonavailability of public transport (US$182 before lockdown versus US$514 during lockdown).

### Discontinuation of TB medicine during complete lockdown period

Approximately 7% of patients in the general population discontinued their TB medicines because of the complete lockdown, and the average number of days of medicine discontinuation was approximately 10 (ranging from 6 to 15 days). This percentage excludes three patients who completely stopped TB medicine during the lockdown period. Patients in tea garden areas did relatively better in treatment adherence (approximately 4% discontinued medicine), which was probably because many tea gardens continued DOT. As the patients lived within the tea garden areas, they managed to continue their medicines even within the complete lockdown period. For both groups of patients, the reasons for medicine discontinuation were either that the TB medicines were not available at the facilities, or that they could not travel because of strict travel restrictions.

## Discussion

This paper presents the financial impact of the COVID-19 nationwide lockdown on TB patient families and health service utilization of TB patients during the lockdown period using primary data collected as part of an ongoing study. Our study found that a significant proportion of the sampled TB patient families had zero income during the complete lockdown period in April–May 2020. This led to huge income loss for the households with TB patients. This loss was slowly restored over the next 3 months (June–August 2020). Eight months after the complete lockdown (January–February 2021), 44% of households in the general population and 37% of those from tea garden areas still suffered from income loss when we compared their income during January–February 2021 with their prelockdown income (February 2020). Our findings corroborate other multiround surveys conducted in India, which also noted a significant proportion of respondents (85%) with reduced monthly incomes or wages during May 2020 compared to the prelockdown period [4, 10].

Before COVID-19, TB disease also had an impact on household income, and income levels were reduced during the intensive phase of TB treatment for all groups of patients compared to before TB. While no households with TB patients in the general population had zero household income before the disease, 17% had zero income during the intensive phase of treatment. This is probably because those TB patients were the only breadwinner of their families, and as they had the disease and could not continue to work, household income became nil. Household income started improving during the continuation phase of treatment, as the patients started joining their work slowly. A similar trend was noticed for families with TB patients in tea garden areas. The COVID-19 nationwide lockdown, however, changed the situation drastically, and a significant proportion of households in both groups had no income during the complete lockdown months (April–May 2020). This is probably because approximately 34–36% of the study participants worked in the informal sector as contractual workers or daily wage earners. Round 3 of the Delhi National Capital Region coronavirus telephone survey also reported that households whose primary source of income was casual wage work or small businesses had suffered the most [10]. Although household income started improving after the end of the strict lockdown, the restoration of income was slow. Income loss during the complete lockdown period was lower among the households in tea garden areas than among the general population, and the restoration of income after the complete lockdown also was better for this group. This is because most of the tea gardens started operations after an average of 20 days of closure during lockdown to avoid significant production loss during the peak season.

Although tea gardens were mostly operational after an average of 20 days of closure during the lockdown, family members among the tea garden residents also work as contractual tea garden workers or daily wage earners in the informal sector. The high percentage of families having income loss even 8 months after the end of the complete lockdown clearly implies that informal sector workers/daily wage earners were the worst affected group, and it also supports the findings of another study indicating that the impact of the complete lockdown has a long-lasting effect on the economy [11].

TB treatment interruption is one of the risk factors for poor treatment outcomes and the possibility of the occurrence of multidrug-resistant TB in the future [12, 13]. A study conducted in one state in India showed that symptom persistence is more common for those who discontinued treatment [12]. The present study supports the above study conclusions. All three patients in the general population who completely stopped their TB medicines during the lockdown had TB again and started treatment as reported during the posttreatment follow-up interviews. Furthermore, almost all patients in the tea garden areas who missed their medicines during the course reported having TB-like symptoms during posttreatment follow-up interviews. Therefore, not only will the lower rate of diagnosis of TB because of the COVID-19 lockdown have an impact on the number of TB cases in the future, the discontinuation of medicine also will impose an additional burden in terms of a greater number of TB cases and maybe multidrug-resistant TB cases.

The following limitations of the study merit comment. First, the current study was carved out of the original study during COVID-19 pandemic. As the study participants of one of the ongoing study states were impacted by the COVID-19 restrictive measures, we estimated the impact for those patients only. We did not have the opportunity to design a study with representative unbiased sample and with adequate power to estimate economic impact of COVID-19 restrictive measures as precisely as we would have liked. Second, the financial impact of COVID-19 was assessed using only household net income loss as an indicator. However, there could be other financial hardships in terms of loans, borrowing, sale/mortgage of assets, use of savings, etc. Those were not considered in this study. The multiround telephone survey reported that approximately 44% of the respondents borrowed during the lockdown to meet their daily necessities [4]. Therefore, it is obvious that the financial impact presented in this study is not comprehensive. Third, self-reported household income may not be accurate, given that a significant proportion of the patients and their families were engaged in the informal sector; however, we assume that the misreporting was consistent for the different time periods. Finally, it might happen that some patients did not disclose that they missed TB drugs because of the lockdown; hence, the numbers reported could be an underestimation, although we expect that as we interacted with the study participants at different intervals for the main study, most of them disclosed the truth.

## Conclusion

This study, a report from the field, corroborates the concern that there were disruptions in TB services during the lockdown in India, and the findings have some implications for India’s TB control program. Discontinuation of medicine because of the lockdown will have an additional burden on the program, with additional TB cases and maybe multidrug-resistant TB cases. Income loss for households and poor restoration of income after the lockdown will likely have an impact on the nutrition of TB patients and families, as it is well recognized that TB disproportionately affects poor people and that there is a bidirectional relationship between undernutrition and TB [14–17]. Although the government of India introduced Nikshay Poshan Yojana in April 2018 to provide nutritional support to all registered TB patients by transferring the benefit directly to the beneficiaries’ bank accounts [18], we found that during the continuation phase of interviews in July–August 2020, approximately 24% of patients in the general population and 42% in tea garden areas did not receive the benefit. In recent years, there has been increased attention given to providing nutritional and socioeconomic support to TB patients. As COVID-19 continues, TB programs must continue to scale these up as core, rather than optional, additions to TB services.

Our study findings highlight the devasting impact of COVID-19 restrictive measures on informal employment. A policy priority must continue to protect informal workers from the economic consequences of such restrictions, including paid sick leave, additional food support, and direct benefit transfers. Alongside ensuring widespread access to COVID-19 vaccines, these policy actions remain pivotal in ensuring the well-being of those who are unfortunate enough to be living with tuberculosis.

## Supplementary Information

**Additional file 1.** Annexure.

## Data Availability

The datasets generated and analysed during the current study are part of an ongoing study and are not publicly available due to the agreement between the study states and the principal investigator. However, these will be available from the corresponding author on reasonable request at the end of the main study.

## Abbreviations

TB: Tuberculosis
DOT: Directly observed treatment

## Footnotes

1 Drug-susceptible TB requires a minimum of six months of treatment, of which the first two months are called the intensive phase and the following four months the continuation phase.
