# Supplementary material for: Impact of COVID-19 restrictive measures on income and health service utilization of tuberculosis patients in India
Source: BMC Infect Dis. 2022 Aug 29;22:711. doi: 10.1186/s12879-022-07681-z (PMC9421110; doi:10.1186/s12879-022-07681-z)
Supplement: Supplementary file 1 — Additional file 1. Annexure. [file 12879_2022_7681_MOESM1_ESM.docx]

*Annexure*

**Impact of COVID-19 restrictive measures on income and health service utilization of tuberculosis patients in India**

*Objective of the ongoing study*

The present study is part of an ongoing study the objective of which is to present the economic burden of TB in India. We collect data to report the journey of the TB patients in India since the onset of TB symptoms till approximately one year posttreatment focusing on out-of-pocket expenses related to TB treatment, proportion of patients experience catastrophic health expenditure (defined as proportion of patients having total cost of TB treatment ≥20% of pre-TB annual household income) and changes in economic status in terms of income and employment and coping strategies. The broad study design is a cohort study with the aim to be representative of different population groups in India.

*Study population*

In India, apart from general population, there are several groups such as people living in urban slum areas, tea garden areas, homeless people, people having improper nutrition, coal mine workers, workers in stone crusher areas who are at higher risk of having TB because they are socially vulnerable and/or clinically high-risk. These high-risk groups were identified for active case finding in the national strategic plan for eliminating TB in India, 2017-2025 [1]. The ongoing study covers representative samples of TB patients from general population as well as from two such high-risk groups, urban slum dwellers and people living in tea garden areas. The high-risk groups were chosen as the treatment seeking behaviour, costs of TB treatment and coping strategies in high-risk groups could be different from general population and different protective measures may be required for them in the process of TB elimination in the country.

As per World Bank 2018 estimates, 35.2% of India’s urban population live in slum areas [2]. Apart from having poor housing condition, sanitation, access to safe drinking water, the urban slum dwellers experience illegality and social exclusion, poorly regulated and ineffective health services, and overall, there is lack of clarity regarding which level of the Indian Government is responsible for protecting and promoting their health [3].

India’s tea industry is one of the largest private employers, and the people living in tea estates entirely rely on estates for employment and other services such as housing, water, health, education [4]. Because of very poor living condition and extremely low wage rate, the families living in tea garden areas are at high-risk of any disease including TB. Their health service utilization pattern, treatment expenses, employment status in pre-, during and post TB period are unknown. Similar for urban slum dwellers. Hence, the main study aims to report the complete journey of the TB patients from general population as well as from tea garden and urban slum areas.

*Sampling strategy*

For the costing study, national representativeness can be achieved by considering a limited number of states selected using an appropriately stratified sampling design. The World Health Organization Study on global AGEing and adult health (SAGE) sampling design was followed in this context [5]. As a first step, 29 states of India were stratified into six levels of development and six geographic locations. A composite development index was constructed using the following indicators at the state level: Infant mortality rate; female literacy rate; full immunization coverage rate; and per capita income. Principal component analysis technique was used to construct the composite index. Quantiles were then used to categorize the states into six levels of development (Table 1). Based on the availability of study budget, Assam, Maharashtra, Tamil Nadu, and West Bengal were selected for this study as these states not only represent different levels of development but also cover different regions of the country. Apart from that, Assam, Tamil Nadu, and West Bengal have substantial land area for tea gardens, and Maharashtra, Tamil Nadu and West Bengal are among the top five states in terms of slum population in the country. TB patients from general population were drawn from all four states.

In the next stage of sampling, 3-5 districts from each state were selected purposively based on the dominance of the high-risk groups. From the sampled study districts, TB units (TUs)^[[1]](#footnote-1)^ were then identified which cater to the study high-risk groups. As all TUs cater general population, there was no specific identification of TUs for covering patients from general population.

*Patient recruitment*

In the next step, from all identified TUs, adult (≥ 18 years) TB patients who were at their intensive phase of treatment^[[2]](#footnote-2)^ during the visit of the study team and gave written informed consent to participate in the study were interviewed. The interviews conducted at 0-2 months of treatment covered their out-of-pocket expenses, income, and employment status, coping strategies from the onset of TB symptoms till the date of interview. They were then followed up during their continuation phase of treatment (5-6 months) and approximately one year posttreatment.

*Sample Size for each group*

Number of drug-susceptible (DS) TB patients required in each group i.e., from general population, tea garden areas and urban slum dwellers was calculated to estimate the mean cost, if the resulting estimate is to fall within 7% of the true estimate with 95% confidence. The following formula was used to derive the number of DS-TB patients that must be sampled in each group

$$n=Z_{1-\alpha/2}^{2}\frac{\sigma^{2}}{(ɛ.{\mu)}^{2}}DEFF$$

where $\sigma$ is the population standard deviation, $\mu$ is the population mean, $\epsilon$ is the relative precision,  $Z_{1-\frac{\alpha}{2}}$ is the $\left( 1-\frac{\alpha}{2} \right)^{th}$ quantile of the standard normal distribution and DEEF is the design effect to account for the cluster sampling design. Design effect helps account for the clustering in costs borne by patients from the same TU. An earlier study conducted in Tamil Nadu state in India [6], reported a mean cost of Indian Rupee (INR) 23,991 along with a standard deviation of INR 12,258 (converted in 2015 prices). Therefore, $\mu$= INR 23,991 and $\sigma$= INR 12,258 were assumed. For the resulting estimate to fall within 7% of the true estimate with 95% confidence, $\epsilon$=7% and $\alpha$=0.05, i.e.  $Z_{1-\frac{\alpha}{2}}=1.96$ were set. For most demographic health surveys, the design effect, which is the ratio of variances under cluster sampling and simple random sampling, comes out to be less than or equal to 2. Therefore, DEFF was set as 2. Based on these specifications, 410 DS-TB patients are required to estimate the mean cost within 7% of the true estimate with 95% confidence accounting for survey design. Considering a 10% loss to follow-up and another 10% non-response, the final sample size of TB patients required will be 512 in each group. This implies that 1,536 DS-TB patients (512 patients in each group x three groups: general population, tea garden areas and slum dwellers) need to be interviewed in total for the main study. 512 patients in each group were sampled from all four states except for patients in tea garden areas who were sampled from three states as the state Maharashtra does not have tea gardens.

**References**

1. Central TB Division, Directorate general of health services, ministry of health and family welfare, Nirman Bhavan, New Delhi. 2017; National strategic plan for tuberculosis elimination, 2017-2025. https://tbcindia.gov.in/WriteReadData/National%20Strategic%20Plan%202017-25.pdf.

2. <https://data.worldbank.org/indicator/EN.POP.SLUM.UR.ZS?locations=IN> accessed April 23, 2022

3. Nolan LB. Slum definitions in urban India: implications for the measurement of health inequalities. Popul Dev Rev 2015; 41(1): 59-84.

4. Rajbangshi PR, Nambiar D. “Who will stand up for us?” the social determinants of health of women tea plantation workers in India. Int J Equity Health 2020; 19: 29. <https://doi.org/10.1186/s12939-020-1147-3>

5. World Health Organization. WHO's Study on global AGEing and adult health (SAGE).

https://www.who.int/data/data-collection-tools/study-on-global-ageing-and-adult-health. Accessed April 23, 2022.

6. John KR, Daley P, Kincler N, Oxlade O, Menzies D. Costs incurred by patients with pulmonary tuberculosis in rural India. Int J Tuberc Lung Dis 2009; 13(10): 1281-1287.

**Table 1: States stratified based on levels of development and regions**

|  | Levels of development | | | | | |
| --- | --- | --- | --- | --- | --- | --- |
| Regions | I (Most developed) | II | III | IV | V | VI (least developed) |
| North | Delhi | Himachal Pradesh, Punjab | Haryana, Uttarakhand |  | Jammu & Kashmir |  |
| Central |  |  |  |  | Chhattisgarh | Madhya Pradesh, Uttar Pradesh,  Rajasthan |
| East |  |  | **West Bengal** |  | Odisha,  Jharkhand | Bihar |
| North-east | Sikkim | Mizoram  Tripura | Manipur | Nagaland  Arunachal Pradesh | Meghalaya | **Assam** |
| West | **Maharashtra**  Goa |  |  | Gujarat |  |  |
| South | Kerala | **Tamil Nadu** | Karnataka | Andhra Pradesh |  |  |

1. One TB unit covers 200,000 population (range 150,000 - 250,000) for rural and urban areas, 100,000 (range 75,000 - 150,000) in hilly, tribal and difficult areas (National Strategic Plan for Tuberculosis: 2017-2025). https://tbcindia.gov.in/WriteReadData/National%20Strategic%20Plan%202017-25.pdf. [↑](#footnote-ref-1)
2. Drug-susceptible TB requires a minimum of six months of treatment, of which the first two months are called the intensive phase and the following four months the continuation phase. [↑](#footnote-ref-2)
